# Glyphosate exposure in early pregnancy and reduced fetal growth: a prospective observational study of high-risk pregnancies

**DOI:** 10.1186/s12940-022-00906-3

**Published:** 2022-10-11

**Authors:** Roy R. Gerona, Jill L. Reiter, Igor Zakharevich, Cathy Proctor, Jun Ying, Robin Mesnage, Michael Antoniou, Paul D. Winchester

**Affiliations:** 1 Department of Gynecology, Obstetrics and Reproductive Sciences, University of California San Francisco, 2340 Sutter St S271, 94115 San Francisco, CA USA; 2 Department of Medical and Molecular Genetics, Indiana University School of Medicine, 410 W. 10th Street, Suite 5000, 46202 Indianapolis, IN USA; 3 Department of Pediatrics/Neonatology, Indiana University School of Medicine, NICU Satellite Franciscan Health, 8111 South Emerson Avenue, 46237 Indianapolis, IN USA; 4 Fay W. Boozman College of Public Health, University of Arkansas for Medical Sciences, 4301 West Markham Street RAHN 3212, 72205 Little Rock, Arkansas, USA; 5 Department of Medical and Molecular Genetics, King’s College London School of Medicine, Guy’s Hospital, Great Maze Pond, 8th Floor, Tower Wing, SE1 9RT London, UK

**Keywords:** Glyphosate, Herbicides, Pregnancy, Fetal growth restriction, Birth weight percentile, Neonatal intensive care unit admission

## Abstract

**Background:**

Prenatal glyphosate (GLY) exposure is associated with adverse reproductive outcomes in animal studies. Little is known about the effects of GLY exposure during pregnancy in the human population. This study aims to establish baseline urine GLY levels in a high-risk and racially diverse pregnancy cohort and to assess the relationship between prenatal GLY exposure and fetal development and birth outcomes.

**Methods:**

Random first trimester urine specimens were collected from high risk pregnant women between 2013 and 2016 as part of the Indiana Pregnancy Environmental Exposures Study (PEES). Demographic and clinical data were abstracted from mother and infant medical records. Urine glyphosate levels were measured as a proxy for GLY exposure and quantified using liquid chromatography-tandem mass spectrometry. Primary outcome variables included gestation-adjusted birth weight percentile (BWT%ile) and neonatal intensive care unit (NICU) admission. Relationships between primary outcome variables and GLY exposure were assessed using univariate and multivariate linear and logistic regression models.

**Results:**

Urine GLY levels above the limit of detection (0.1 ng/mL) were found in 186 of 187 (99%) pregnant women. Further analyses were limited to 155 pregnant women with singleton live births. The mean age of participants was 29 years, and the majority were non-Hispanic white (70%) or non-Hispanic Black (21%). The mean (± SD) urine GLY level was 3.33 ± 1.67 ng/mL. Newborn BWT%iles were negatively related to GLY (adjusted slope ± SE = -0.032 + 0.014, p = 0.023). Infants born to women living outside of Indiana’s large central metropolitan area were more likely to have a lower BWT%ile associated with mother’s first trimester GLY levels (slope ± SE = -0.064 ± 0.024, p = 0.007). The adjusted odds ratio for NICU admission and maternal GLY levels was 1.16 (95% CI: 0.90, 1.67, p = 0.233).

**Conclusion:**

GLY was found in 99% of pregnant women in this Midwestern cohort. Higher maternal GLY levels in the first trimester were associated with lower BWT%iles and higher NICU admission risk. The results warrant further investigation on the effects of GLY exposure in human pregnancies in larger population studies.

**Supplementary Information:**

The online version contains supplementary material available at 10.1186/s12940-022-00906-3.

## Background

Glyphosate (GLY, N-(phosphonomethyl) glycine) is the most widely used broad spectrum herbicide in agricultural, commercial, and residential areas in the United States. GLY is the active ingredient in commercial formulations of glyphosate-based herbicides (GBHs) that are commonly used on corn, wheat, soy and cotton fields to manage invasive weeds and grasses. Roundup was the first GBH to enter the market in the 1970s; however, over 750 GBHs currently exist for use in the US and worldwide. The development of genetically-engineered GLY-tolerant crops and preharvest desiccant use of GLY in whole grain crops (e.g. oats, wheat) has increased GBH agricultural use 35-fold since the 1970s from 7.8 million pounds to 276 million pounds in 2014 [1]. GLY is also widely used commercially and residentially in areas such as gardens, public parks, school grounds, playing fields, and along roads and railway tracks. Consequently, GLY exposure is frequent in both humans and animals.

As an herbicide, the primary mode of GLY action is inhibiting 5-enolpyruvylshikimate-3-phosphate synthase (EPSPS) of the shikimate pathway, which is involved in the biosynthesis of aromatic amino acids in plants. Inhibition of EPSPS leads to a shortage in essential aromatic amino acids resulting in plant death [2]. The absence of the shikimate pathway in vertebrates and a rapid elimination of GLY in mammals led to the assumption that humans and other mammals should not experience significant toxicity from GLY exposure [3].

GLY, an organophosphorus compound has been extensively studied and many potentially toxic effects have been reported including inhibition of acetylcholinesterase activity in animal models [4, 5]. Additionally, low doses of GLY and GBHs increase oxidative stress and inhibit mitochondrial bioenergetics in various animal models [6].

*In utero* exposure to GLY has been linked to birth defects, fetal loss, and decreased reproductive function in animals such as chickens, frogs, and other mammals [7–9]. In mice, prenatal GLY exposure at the acceptable daily intake dose range resulted in lower sperm counts and decreased testosterone in male offspring [10]. In addition, GBH exposure in pregnant rats induced adverse effects in second generation offspring, including a higher incidence of small for gestational age (SGA) fetuses and delayed growth of F2 pups [11]. Moreover, GBHs administered to rats during pregnancy and through adulthood caused significant androgen effects on genital development in both male and female offspring and exposure to Roundup was associated with delayed onset of puberty and higher testosterone levels and free thyroxine index in females [11]. Another emerging line of evidence suggests that some of the toxic effects of prenatal GLY exposure are caused by it changing the composition of gut microbiota in juvenile offspring [12].

GLY and GBHs also give rise to epigenetic changes, particularly alterations in DNA methylation patterns, that can be passed on to future generations. For example, transgenerational epigenetic effects were demonstrated in rats [13, 14]. In these studies, only pregnant F0 females were administered 25 mg per kg body weight per day (mg/kg bw/d) GLY intraperitoneally from day 8 to day 14 of gestation and GLY-naïve offspring were bred on to the F3 generation. Although F0 animals showed no adverse effects from GLY treatment, there was a dramatic increase in pathologies in the F2 and, crucially, the F3 transgenerational offspring such as obesity, disease of the kidney, ovary, and prostate, as well as higher death rates of late-stage pregnant F2 females or their pups at, or immediately after, birth. These pathologies were correlated with differential DNA methylation regions in sperm, some of which were associated with genes previously shown to be involved in the observed pathologies [13, 14]. Although, these studies provided compelling evidence for GLY-induced transgenerational effects, the implications for human health remain unknown due to the relatively high GLY doses and non-physiological route of administration that were used.

Existing observational studies in humans are limited and frequently lack direct measurements of GLY; as a result, evidence is mixed as to whether current levels of occupational and environmental GLY exposures represent a risk for human development and reproduction. For example, the Ontario Farm Family Health Study (OFFHS) found a significant association between preconception exposure to GBHs and increased risk of spontaneous abortion [15]. In addition, ambient GLY exposure within 2000 m of a pregnant woman’s residence in California increased the risk of autism spectrum disorder (ASD), as well as the risk of ASD with intellectual disability in the offspring [16]. On the other hand, the Agricultural Health Study did not find an association between maternal GLY exposure and birth weight, although birth weight was not corrected for gestation in their study [17].

Our earlier prospective Indiana birth cohort study found that 93% of low-risk pregnant women had detectable urine GLY levels and that higher urine GLY levels were associated with shortened pregnancy length. No association was found with fetal growth as referenced by BWT%ile. A sub-analysis based upon self-reported data suggested that food and beverage consumption may be a potential exposure pathway, although GLY was not detected in residential tap water samples [18]. A second US cohort of low-risk pregnant women, The Infant Development and the Environment Study (TIDES), also demonstrated that higher urine GLY levels were associated with shortened pregnancy gestation [19]. A nested case-control study of pregnant women in Puerto Rico found that preterm birth (a dichotomous measure of shortened gestation) was significantly correlated with both urine GLY and its presumptive metabolite aminomethylphosphonic acid (AMPA) measured at 26 weeks gestation [20]. Thus, these new findings provide evidence that, in selected populations, GLY may shorten the length of pregnancy.

In the U.S., 8% of the pregnant population have high-risk pregnancies, that is pregnancy complications that put the health or life of a woman or her fetus at risk. Such high-risk pregnancy complications may involve medical and obstetrical issues like preexisting diabetes, chronic high blood pressure, preeclampsia, preterm labor, and other complex medical conditions that affect pregnancy. Complications may also involve unexpected problems during pregnancy, such as early labor, bleeding or high blood pressure or babies who may have birth defects or growth problems. Some complications are inter-related such as hypertension which increases the woman’s risk of stroke or heart attack, often decreases fetal growth (intrauterine growth restriction), increases the risk of abruption of the placenta, and contributes to early labor and preterm birth. The extent to which GLY exposure may impact pregnancies and contribute to adverse outcomes is not well understood.

As our previous low-risk cohort found an association with GLY and shortened pregnancy, we sought to conduct a larger study of more diverse women with higher risk pregnancies living across the state of Indiana. We collected urine from pregnant women attending a University-based Maternal-Fetal Medicine Specialty Obstetrics Clinic that specializes in the care of mothers and fetuses during high-risk pregnancies, with the chief aims to: (1) Establish the prevalence of detectable urine GLY in high-risk pregnancies, (2) Correlate urine GLY levels and maternal characteristics and co-morbidities in high-risk pregnancies, and (3) Investigate correlations between first trimester maternal GLY levels and fetal growth indicated by birth weight percentiles and risk of NICU admission.

## Methods

### Study participants

A subset of women in early pregnancies were identified from our Indiana Pregnancy Environmental Exposures Study (PEES) Cohort which consists of 853 high risk pregnancies from 822 women with over 3000 urine samples. Inclusion criteria for the PEES cohort required a living conceptus at the time of the sample collection in women who were at least 18 years of age. Discarded random urine samples were collected during the course of prenatal care from the Maternal-Fetal Medicine Specialty Obstetrics Clinic at Indiana University Hospital between the years 2013 to 2017. This clinic offers specialty obstetrics care for a wide variety of maternal or fetal conditions considered to be high-risk, including diabetes, recurrent pregnancy loss, prior stillbirth, prior preterm birth, cervical insufficiency, chronic or gestational hypertension, cardiac or other diseases, substance abuse, fetal malformations, fetal growth problems, and alloimmunization, etc. The racial/ethnic makeup of the PEES cohort is 77% White, 19% Black, 2% Asian, and 1% Hispanic. In addition, women from 68 of Indiana’s 93 counties are represented with over half the subjects living outside Marion County (Indianapolis). All PEES study samples were transported to the processing laboratory at the end of the clinic day, assigned a unique study ID, and stored frozen at -20 °C; long-term storage was at -80 °C and maintained in the Indiana Clinical and Translational Sciences Institute (CTSI) Specimen Storage Facility (SSF). The research protocols were approved by the Indiana University Institutional Review Board with waivers of informed consent and authorization for the use of protected health information as it involved no more than minimal risk of loss of privacy to the subject and no participant contact was attempted.

For the present study, all high risk pregnancies with a first trimester (< 14 weeks) urine sample (≥ 1 mL) that had not undergone a freeze-thaw cycle were initially selected for urine GLY analysis (n = 215). One batch of 28 samples did not pass the analytical laboratory’s quality control cycle criteria and were not analyzed further. Statistical analysis of birth outcomes (BWT%ile and NICU admission) was conducted on 155 first trimester samples, which also excluded 32 pregnancies with multiple gestations, fetal loss, and unverified deliveries. The study population consisted of 155 pregnancies resulting in 155 singleton live births from 150 women.

### Abstraction of medical records

Study data were abstracted for each pregnancy from electronic medical records following newborn delivery and coded information was stored in a secure research electronic data capture (REDCap) database. All medical records were collected without prior knowledge of urine GLY results. Each maternal record was reviewed for pre-pregnancy factors, pregnancy risk factors, gestational length, and fetal growth indicators as well as neonatal outcomes. Gestational length was calculated in days based on last menstrual period and obstetrical adjustment by first ultrasound. *Pre-pregnancy factors* included maternal age, parity, race/ethnicity, education, employment, insurance, marital status, county and state of residence, pre-existing health conditions and substance use. Maternal county and state of residence were used to construct metro classifications for each participant based upon the 2013 National Center for Health Statistics (NCHS) Urban-Rural Classification Scheme for Counties [21]. In Indiana, only Marion County is defined as a large central metro area, a county population of 1 million or more with a high population density per square mile and a low percent of exurban/rural population. *Pregnancy risk factors* collected for this study were medical diagnoses obtained following perinatal screenings for healthcare purposes that were documented in electronic medical records. Study data included on-going maternal diseases, hypertensive disorders of pregnancy (chronic hypertension, pregnancy induced hypertension, pre-eclampsia, and eclampsia), diabetes (pre-existing and gestational), substance use (drug, alcohol, tobacco, caffeine use), stress, body mass index (BMI) at delivery, duration of pregnancy, and route of delivery. For purposes of this study, the American College of Obstetrics and Gynecology (ACOG) definitions for hypertensive disorders of pregnancy and diabetes were used. Maternal stress was defined as high stress during pregnancy that was self-reported during obstetrical evaluation as noted in the patient’s electronic medical record. *Neonatal factors* included race/ethnicity, sex, fetal growth indicators such as birth weight and gestational age, NICU transitional care and admission, as well as neonatal diagnoses such as congenital anomalies, neonatal abstinence, respiratory distress, and prematurity. To standardize fetal growth across gestations, gestational age and birth weight were used to calculate a BWT%ile for each liveborn infant using Fenton 2013 Growth Curves for each sex [22].

#### Analytical method

All urine samples were de-identified and coded prior to shipping to the University of California San Francisco Clinical Toxicology and Environmental Biomonitoring Laboratory for GLY analysis. Urine GLY was measured by standard addition using an Agilent LC 1260 (Agilent Technologies Inc, Santa Clara, CA)-AB Sciex 5500 Triple Quadrupole MS (SCIEX, Redwood City, CA). Isocratic elution chromatography was performed using an Obelisc-N mixed-mode column (2.1 × 100 mm, 5 μm) that was maintained at 40 °C. A 25 µL sample was injected into the column and GLY was eluted using a mobile phase of 1% formic acid in bisphenol-A free water at a flow rate of 1 mL/min and a total run time of 6 min.

Mass spectral analysis was performed using an electrospray ionization source operated in negative mode. The parameters used for ionization included curtain gas, 20 psi; collision gas, 9 psi; ion spray voltage, -4500 V; temperature, 700 °C: and ion source gas, 60 psi. GLY was monitored using two transitions: 168.1–62.9 m/z (quantifier) and 168.1–81.0 m/z (qualifier). We used 2-^13^C,^15^N-GLY as an internal standard that was monitored using the 169.4–63.0 m/z transition. Quantitative analysis of GLY was done by isotope dilution method.

Each batch of samples was injected in duplicate. Procedural quality control (QC) materials and procedural blanks were run along with the samples at the start, middle, and end of each run. Two QC materials were used at low and high concentrations. To accept the results of a batch run, QC materials measurements must be within 20% of their target values. GLY identification from total ion chromatograms was evaluated using AB Sciex Analyst v2.1 software while quantification was processed using AB Sciex MultiQuant v2.02 software.

All measured urine samples were corrected for specific gravity in our analysis for differences in urine dilution. The established limits of quantification (LOQ) and limits of detection (LOD) for GLY in urine were 0.5 and 0.1 ng/mL, respectively [23, 24].

### Statistical analyses

Primary clinical outcome measures were the numerical variable of birth weight adjusted for gestation, BWT%ile, and a binary variable of NICU admission during newborn hospitalization. The continuous measure of exposure, urine GLY (ng/mL) was considered a major independent variable of interest. Socio-demographics, substance use, and other pregnancy and delivery-related risk factors were categorized and used as controlling covariates and moderators in the analysis. The primary biostatistical methods were a linear regression model to assess the relationship between BWT%ile and GLY and a logistic regression model to assess the relationship between NICU admission risk and GLY. Both adjusted and unadjusted methods were used in the statistical models. The unadjusted or univariate models used GLY as the only independent variable in the analysis, while the adjusted or multivariate models included other controlling covariates along with GLY as the independent variables. In order to assess if the relationships of the dependent variables to the GLY level were moderated by a controlling covariate, we used linear and logistic regression models but added GLY, the controlling covariate or moderator, and their interaction as independent variables in the analysis. A moderator was considered significant if the p-value of the interaction term was less than 0.05. In addition, slopes of GLY under subgroups stratified by the moderator were estimated from the linear regression model or logistic regression model and used to assess the relationships under the subgroups. Finally, one-way fixed effect models (or one-way ANOVA models) were used to assess the associations of GLY to the categorical controlling covariates. Statistical models were computed using SAS 9.4 software (SAS, Cary, NC). P values less than 0.05 were considered statistically significant.

## Results

All pregnancies with a first-trimester (< 14 weeks) urine sample (≥ 1 mL) that had not undergone a freeze-thaw cycle in PEES were initially selected for this study (n = 215). One batch of 28 samples did not meet laboratory quality control criteria. Of the remaining 187 samples, 186 (99.5%) had GLY levels  > LOD. To determine the relationship between first trimester GLY urine measurements and outcomes of singleton newborns, 32 pregnancies were excluded (20 with fetal losses, 9 with multiple gestations, 2 unverified deliveries, and 1 with GLY < LOD). Thus, the subset study cohort included 155 pregnancies with singleton liveborn infants. The study population included pregnant women from 32 Indiana counties and one adjacent Illinois county; 57% of the participants lived in Indiana’s large central metropolitan area. The mean maternal age was 29 years (range 18–45 years). The mean pregnancy length was 37.9 weeks (mean ± SD, 265.2 ± 12.7 days). Characteristics of the study participants and their newborns along with the maternal first trimester urine GLY levels are summarized in Table 1.

**Table 1:** Mother and infant characteristics and first trimester glyphosate (GLY)

Characteristic	Group	Count (%)	GLY	
		**(Total N = 155)**	**Mean ± SE**	**p-value** ^**a**^
***Maternal***				
Age	≤ 25 years	49 (31.6)	3.41 ± 0.24	0.605
	25–35 years	76 (49.0)	3.38 ± 0.19	
	> 35 years	30 (19.4)	3.05 ± 0.31	
Race/Ethnicity	White	109 (70.3)	3.36 ± 0.16	0.866
	Black	32 (20.7)	3.37 ± 0.30	
	Hispanic	7 (4.5)	3.18 ± 0.64	
	Other	7 (4.5)	2.82 ± 0.64	
Marriage Status	Not Married	64 (41.3)	3.20 ± 0.21	0.418
	Married/Cohabitation	91 (58.7)	3.42 ± 0.18	
Education	<High School	7 (4.5)	5.16 ± 0.61	**0.003**
	High School	39 (25.2)	3.07 ± 0.26	
	>High School	78 (50.3)	3.10 ± 0.18	
	Unknown	31 (20.0)	3.80 ± 0.29	
Employment	Not Employed	73 (47.1)	3.36 ± 0.20	0.834
	Employed	82 (52.9)	3.30 ± 0.19	
Living in Large Central Metro	No	66 (42.6)	3.25 ± 0.21	0.607
	Yes	89 (57.4)	3.39 ± 0.18	
Tobacco Use	No	104 (67.1)	3.14 ± 0.16	0.051
	Yes	51 (32.9)	3.70 ± 0.23	
Caffeine Use	No	59 (38.1)	3.73 ± 0.21	**0.018**
	Yes	96 (61.9)	3.08 ± 0.17	
Alcohol Use	No	136 (87.4)	3.31 ± 0.14	0.746
	Yes	19 (12.3)	3.45 ± 0.40	
Opioid, THC, or Polysubstance Use	No	105 (67.7)	3.24 ± 0.16	0.340
	Yes	50 (32.3)	3.51 ± 0.24	
High Stress	No	114 (73.6)	3.26 ± 0.16	0.381
	Yes	41 (26.5)	3.52 ± 0.26	
Hypertensive Disorder(s)	No	98 (63.2)	3.46 ± 0.17	0.185
	Yes	57 (36.8)	3.09 ± 0.22	
Diabetes	No	115 (74.2)	3.50 ± 0.15	**0.030**
	Yes	40 (25.8)	2.83 ± 0.26	
Delivery BMI	≤ 25	21 (13.6)	2.69 ± 0.36	0.210
	25–30	40 (25.8)	3.63 ± 0.26	
	30–35	50 (32.3)	3.52 ± 0.23	
	35–40	25 (16.1)	3.02 ± 0.33	
	> 40	19 (12.3)	3.29 ± 0.38	
C-Section	No	80 (51.6)	3.36 ± 0.19	0.810
	Yes	75 (48.4)	3.29 ± 0.19	
***Infant***				
Sex	Female	78 (50.3)	3.38 ± 0.19	0.685
	Male	77 (49.7)	3.27 ± 0.19	
Birth Defects	No	135 (87.1)	3.38 ± 0.14	0.305
	Yes	20 (12.9)	2.97 ± 0.37	
Preterm Birth	< 37 weeks	32 (20.7)	3.43 ± 0.30	0.688
	≥ 37 weeks	123 (73.4)	3.30 ± 0.15	
Neonatal Abstinence Syndrome (NAS)	No	131 (84.5)	3.22 ± 0.14	0.067
	Yes	24 (15.5)	3.90 ± 0.34	

^a^ P-values were obtained from one-way fixed effect models

### Associations between maternal characteristics and GLY levels

Mean (± SD) GLY level was 3.33 ± 1.67 ng/mL (range 1.02–10.31 ng/mL) for the eligible study population (n = 155). Comparatively, the mean (± SD) GLY level for the excluded individuals (n = 32) was 2.86 ± 1.41 ng/mL (range 0.10–6.89 ng/mL). No statistically significant differences were found between the two groups based upon both parametric and non-parametric tests (Table 2). In a sensitivity analysis we also added the single participant whose GLY was < LOD using the LOD as the GLY level. The findings were not changed and thus, the single participant with a GLY level  < LOD was excluded from the final 155 cases.

**Table 2:** Summary of urine glyphosate (GLY) levels based upon eligibility criteria

Study Population	N	Mean	Median	SD	Min	Max	P-value	
							T-test	Wilcoxon rank sum test
Eligible/Included	155	3.33	3.08	1.67	1.02	10.31	-	-
Not Eligible/Excluded (undetectable GLY sample not removed)	31	2.95	2.95	1.34	1.00	6.89	0.241	0.339
Not Eligible/Excluded (undetectable GLY sample removed)	32	2.86	2.93	1.41	0.10	6.89	0.144	0.224

No differences in GLY levels were found based on maternal age, race, or residence in a large central metro area; however, GLY appears to be associated with maternal education. In particular, participants with less than a high school degree had significantly higher urine GLY levels compared to groups with a high school degree or higher (mean ± SE, 5.16 ± 0.61 vs. 3.07 ± 0.26 ng/mL, p = 0.003). Though higher maternal GLY levels were found in lower educated women there were only seven women in the < High School group, two with urinary GLY levels of 8.77 ng/mL and 10.31 ng/mL, the highest among all participants. Statistical significance was not found after these two individuals were removed from analysis (p = 0.146).

Higher GLY levels were also found in pregnant women who used tobacco during pregnancy (p = 0.051), while significantly lower GLY levels were found in participants who consumed caffeine during pregnancy (p = 0.018). Although not statistically significant, higher GLY levels were found in participants whose newborns were treated for neonatal abstinence syndrome (p = 0.067); however, there were no differences in GLY levels between participants who did or did not use opioids, cannabis, or polysubstances (multiple drug use). Paradoxically, significantly lower GLY levels were found in pregnant women with diabetes (p = 0.03), while there were no differences in GLY levels based on delivery BMI.

### Relationship between maternal urine GLY and newborn birth weight

**Fig. 1:**
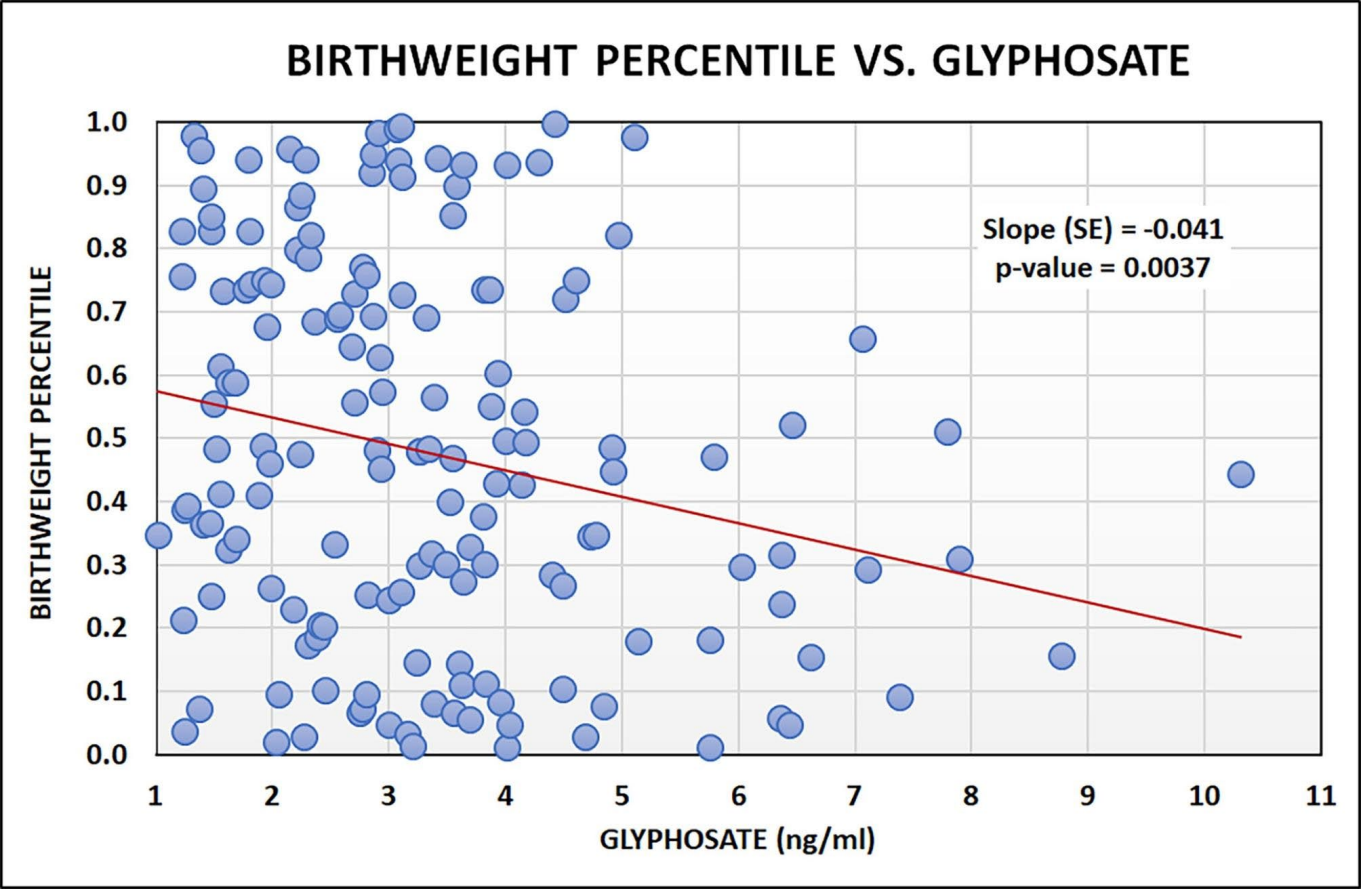
Infant birth weight percentile vs. first trimester urine glyphosate level Scatter plot of maternal urine glyphosate measures in the first trimester of pregnancy and newborn birthweight adjusted for gestation. Each point represents one mother-infant pair (n = 155). Red line indicates the slope calculated from the linear regression model. Each one-unit increase in GLY resulted in a 4.1% drop in birth weight percentile.

To investigate whether maternal GLY levels in the first trimester of pregnancy were related to fetal growth, we used linear regression models to assess the relationship between GLY and newborn birth weight adjusted for gestation (BWT%ile). Mean (± SD) BWT%ile for the singleton newborns was 47.7 ± 30.1. We found that BWT%ile was negatively related to GLY (Slope ± SE = -0.041 ± 0.014, p = 0.004) (Fig. 1). This negative relationship remained significant after controlling for social demographics and geographic characteristics (maternal age, race, education, employment, marriage, residence, and infant sex), health characteristics (maternal hypertension, diabetes, and delivery BMI), and behavior characteristics (tobacco, alcohol, caffeine, opioid/THC or illicit polysubstance use) in the adjusted model (Slope ± SE = -0.032 ± 0.014, p = 0.023) (Table 3). Tobacco use, alcohol use, opioid/THC or polysubstance use, maternal hypertension, residence, and infant sex were found significant moderators when they were interacted with the relationship between GLY and BWT%ile individually. In particular, when comparing the relationship between GLY and BWT%ile in sub-groups, there was a stronger negative relationship in participants using tobacco (Slope ± SE = -0.046 ± 0.019, p = 0.016) compared to those who did not smoke (Slope ± SE = -0.027 ± 0.022, p = 0.208). Similar findings were observed in participants with opioid/THC or illicit polysubstance use compared to those with no use of these substances. On the other hand, the negative relationship between GLY and BWT%ile was weaker for participants who used alcohol or had hypertension. While we did not observe significant differences in GLY levels between participants living in or outside of Indiana’s large central metropolitan area (Table 1), we found that women living outside of Indiana’s large central metropolitan area were more likely to have a negative relationship between BWT%ile and GLY (Table 3).

We also noted that BWT%ile in this cohort was significantly lower in non-Hispanic black than in non-Hispanic white pregnancies (25.7 vs. 53.8, p < 0.001) and that the negative relationship between GLY and BWT%ile was strongest for male infants. However, the size of this study cohort was too small to further investigate the relationship between first trimester urine GLY and BWT%ile in each maternal race and infant sex.

Additional analysis was performed to further assess the relationship among the dependent variable (BWT%iles), the independent variable (GLY) and maternal and infant characteristics listed in Table 1 (i.e., alcohol use, tobacco use, substance use, hypertensive disorders of pregnancy, diabetes, infant sex, etc.) (See Additional File 1, Supplemental Table 1).

In a sensitivity analysis we used GLY after adjusting for gestational age using a regression method and repeated the statistical analysis using this adjusted GLY instead of the unadjusted GLY. The findings were the same as those reported in our results. Hence those results using adjusted GLY were not reported. In another sensitivity analysis we also adjusted GLY for gestational age at time of urine collection (4–13 weeks); however, this analysis did not change the findings (data not shown).

**Table 3:** Relationship between first trimester urine glyphosate (GLY) and gestation-adjusted birth weight (BWT%ile)

Characteristic	Group	GLY	
		**Slope ± SE**	**p-value**
All (unadjusted)	-	-0.041 ± 0.014	**0.004**
All (adjusted) ^a^	-	-0.032 + 0.014	**0.023**
Tobacco Use ^b^	No	-0.027 ± 0.022	0.208
	Yes	-0.046 ± 0.019	**0.016**
Alcohol Use ^b^	No	-0.049 ± 0.015	**0.001**
	Yes	0.013 ± 0.043	0.760
Opioid, THC or Polysubstance Use ^b^	No	-0.025 ± 0.021	0.224
	Yes	-0.056 ± 0.019	**0.004**
Hypertensive Disorder(s) ^b^	No	-0.045 ± 0.016	**0.005**
	Yes	-0.017 ± 0.031	0.598
Living in Large Central Metro ^b^	No	-0.064 ± 0.024	**0.007**
	Yes	-0.028 ± 0.017	0.108
Infant Sex ^b^	Female	-0.023 ± 0.018	0.204
	Male	-0.069 ± 0.022	**0.002**

^a^ The All (adjusted) method controlled for covariates of tobacco use, alcohol use, opioid/THC or polysubstance use, hypertension, living in large central metro area, and infant sex

^b^ A significant moderator with p-value < 0.05 in the interaction term

### Relationship between maternal urine GLY and newborn admission to intensive care

We used logistic regression models to investigate whether maternal GLY levels in the first trimester of pregnancy were related to the risk of their newborn being admitted to the NICU. Sixty-nine out of 155 infants (44.5%) from this high-risk obstetrical cohort were admitted to the NICU after birth. The odds ratio (OR) of NICU admission corresponding to one unit increase of GLY was 1.21 (95% CI: 0.99, 1.38, p = 0.069) (Table 4). When the adjusted model was used, the positive relationship was also not significant (p = 0.233). However, significant relationships were observed in subset analyses among significant moderators of alcohol use, opioid/THC or polysubstance use, maternal hypertension, residence, and infant sex. Significant positive relationships between NICU admission and GLY was found in participants with no use of alcohol or opioid/THC or polysubstance use, no hypertension, and those living in Indiana’s large central metropolitan area.

**Table 4:** Relationship between first trimester glyphosate (GLY) and neonatal intensive care unit (NICU) admission

Characteristic	Group	Dependent Variable = NICU Admit		
		**Independent Variable = GLY**		
		**Slope ± SE**	**OR (95% CI)**	**p-value**
All (Unadjusted)	-	0.188 ± 0.103	1.21 (0.99, 1.38)	0.069
All (Adjusted) ^a^	-	0.145 ± 0.130	1.16 (0.90, 1.67)	0.233
Tobacco Use	No	0.260 ± 0.155	1.30 (0.96, 1.62)	0.093
	Yes	0.039 ± 0.152	1.04 (0.77, 3.42)	0.800
Alcohol Use ^b^	No	0.247 ± 0.115	1.28 (1.02, 1.37)	**0.032**
	Yes	-0.204 ± 0.327	0.82 (0.43, 1.99)	0.533
Opioid, THC, or Polysubstance Use ^b^	No	0.323 ± 0.152	1.38 (1.03, 1.51)	**0.034**
	Yes	0.038 ± 0.145	1.04 (0.78, 3.35)	0.795
High Stress	No	0.248 ± 0.128	1.28 (1.00, 1.46)	0.053
	Yes	0.030 ± 0.184	1.03 (0.72, 4.62)	0.872
Hypertensive Disorder(s) ^b^	No	0.296 ± 0.125	1.34 (1.05, 1.38)	**0.017**
	Yes	-0.128 ± 0.220	0.89 (0.58, 2.33)	0.560
Living in Large Central Metro ^b^	No	0.000 ± 0.164	1.00 (0.73, 5.78)	0.998
	Yes	0.314 ± 0.142	1.37 (1.04, 1.46)	**0.026**
Infant Sex	Female	0.169 ± 0.131	1.18 (0.92, 1.69)	0.196
	Male	0.226 ± 0.166	1.25 (0.91, 1.78)	0.173

^a^ The All (adjusted) method controlled for covariates of tobacco use, alcohol use, opioid/THC or polysubstance use, high stress, hypertension, living in central metro and infant sex

^b^ A significant moderator with a p-value < 0.05 in the interaction term

Additional analysis was performed to further assess the relationships among the dependent variables (NICU Admit), the independent variable (GLY) and maternal and infant characteristics listed in Table 1 (i.e., alcohol use, tobacco use, substance use, hypertensive disorders of pregnancy, diabetes, infant sex, etc.) (See Additional File 1, Supplemental Table 1).

## Discussion

The major finding of this study is that GLY was detectable in the urine of 99% (186/187) of midwestern US women with high risk pregnancies in their first trimester. A mean urine GLY level of 3.3 ng/mL was found in the pregnancies resulting in a singleton live birth (n = 155). These findings are in close agreement with our previous study that found a mean urine GLY level of 3.4 ng/mL in 93% (66/71) of Indiana women with low-risk pregnancies [18]. Participants in our earlier study were predominantly Caucasian (94%) and lived in central Indiana. Therefore, the high incidence of GLY detection reported herein is not likely related to the high-risk nature of the pregnancies, but rather to the widespread environmental use of GLY.

Unexpectedly, this study found lower urine GLY levels in women with any form of diabetes (type I, type II, or gestational) (p = 0.03) and in women who consumed caffeine (p = 0.018) during pregnancy (Table 2). The reason for this finding is unclear, but possible explanations could include potential dietary changes in pregnant women who are being treated for diabetes and physiological consequences of polyuria, which is seen more often with diabetes and caffeine consumption. This finding is in contrast to the association of maternal GLY with diabetes and caffeine in our previous low-risk cohort, which included fewer participants and only six women with diabetes; therefore, further study is needed to clarify the true association. Whereas GLY was correlated with shortened pregnancy length in our previous investigation of low risk pregnancies [18], that finding was not replicated in this study, most likely because of the many co-morbidities of high-risk pregnancies that lead to an obstetrical decision to deliver the baby at an earlier gestation time point.

To our knowledge, there are no animal or human studies which correlate prenatal glyphosate exposure and fetal growth or BWT%ile. An Agriculture Health Study using self-report as a measure of GLY exposure in pregnancy did not find a statistically significant association with birth weight; however, birth weight percentile was not measured, and most participants were applicators with multiple pesticide exposures [17]. Our previous study measuring birth weight percentiles in low-risk pregnancies did not find a statistically significant association with GLY. Though we did find a trend towards lower birth weight percentiles, our data suggested that a larger sample size might be warranted [18]. An animal study found perinatal exposure to GLY did not alter the weight of the exposed animals when compared with the control groups. Exposed rodents weren’t weighed until PND90 which is considered adulthood. Moreover, that study did not examine birth weight percentiles or birth weights [25]. Another animal study found weaning weights to be significantly lower in rodents exposed to glyphosate in utero. The findings further showed increased rates of adult obesity in F2 and F3 descendants of the GLY exposed lineage [26].

Our finding that higher urine GLY levels in pregnancy was associated with lower BWT%iles is compatible with a rodent study, in which smaller birth weights were observed although litter effects were not factored [27]. GLY exposure of our cohort of pregnant women in the first trimester approximates to the time of exposure of rats showing marked fetal epigenetic (DNA methylation) alterations with subsequent transgenerational effects [13, 14]. Thus, our observation of first trimester GLY exposure may have long term health consequences^44^.

A consensus group expressed worldwide concern to scientists, physicians, and regulatory officials about the unanticipated risks to human health and the environment arising from the increasing use of GBHs. They suggested that scientifically up-to-date human studies based on biomonitoring should be prioritized by US government regulatory agencies [28]. When a new chemical is approved for release into the environment such that it is found in every sector (rain, dust, water, air, food, and beverages) and in 94–99% of pregnant women, it is vital that extensive safety measures be undertaken. Indirect causes of toxicity such as those linking GLY with altered microbiome and perhaps such adverse outcomes as autism spectrum disorder, are examples of potential harm, which have not been considered in regulatory agency decisions [29]. It has recently been shown that GLY and a GBH can inhibit the shikimate pathway of bacteria present in the rat gut microbiome with possible health consequences [30]. Furthermore, bioinformatics scrutiny of the human microbiome database, particularly that pertaining to the gut, revealed many bacterial species that would be susceptible to inhibition of their innate shikimate pathway by a GBH [31]. With the rodent models suggesting that multiple generations may be at risk it is imperative that human studies are funded, which can establish long-term risks more unequivocally than these observational studies [13, 26].

An important finding in our study is that BWT%ile was significantly lower in non-Hispanic black compared to non-Hispanic white pregnancies, which is consistent with previous reports in U.S. infants [32]. Unfortunately, the size of our study cohort was too small to further investigate the relationship between first trimester urine GLY and BWT%ile in each maternal race and infant sex. Another limitation of our study is that fetal loss, ethnicity, and maternal co-morbidities were not controlled. In addition, our study participants were not selected other than prospectively attending a Maternal-Fetal Medicine Specialty Obstetrics Clinic for high-risk pregnancies. Because only 6–8% of all pregnancies in the US are estimated to be categorized as high risk, our findings cannot be easily generalized to low-risk pregnancies.

Our study was also underpowered to establish whether GLY is a risk for fetal death or birth defects. Only in pregnancies using assisted reproductive technology, where the day of conception is known, would a full accounting of pregnancy loss related to GLY be possible. Observational studies such as this one, are helpful in defining the scope of exposure (99%) and the correlations with shortened gestation, lower BWT%tile, and increased NICU admission rates, but cannot prove cause and effect. Additional human studies with much larger numbers will be needed to further support our preliminary findings, since no prospective, double-blind randomized study could be ethically done in humans. While animal models do provide evidence that GLY exposure in pregnancy results in reduced fetal growth and weaning weights [33], whether GLY exposure negatively impacts fetal growth or NICU admission risk in humans is only a correlation thus far.

Sources of GLY exposure were not investigated in this study. One study affirmed that drinking water was not a main source of GLY exposure and that higher GLY in those residing in rural areas suggest that some GLY exposure occurs in relation to farming activity [18]. Most GLY exposure likely occurs through consumption of food and beverages (most of which have been found to contain GLY residues) and to some extent through air and precipitation [34–36], but we cannot rule out caffeine, tobacco, and opioids as other potential sources of exposure. In addition, this study did not investigate GBH co-formulant surfactants in urine samples, which could also be contributing to toxicity [1, 37]. A large body of published results have shown that GBHs are more toxic than GLY alone via different mechanisms [2]. For example, work by Mesnage and colleagues found that Roundup was the most toxic of all herbicides and insecticides tested and was found to be 125 times more toxic than GLY in human liver HepG2, human embryonic kidney HEK293, and human placental JEG3 cell lines [38]. The recent development of an assay for detecting GBH surfactants in the urine of rats exposed to a GBH, paves the way for future studies in humans [39].

AMPA is not considered a human metabolite of GLY but is rather likely to be a product of GLY degradation by soil microbes [40–42]. AMPA is found in food residues and has been considered to be of similar toxicity to glyphosate [40–42]. At the onset of this study, reliable assays for AMPA were not available and therefore not measured. However, more recent studies suggest that AMPA is associated with higher biomarkers for oxidative stress than GLY [43]. The combined levels of GLY and AMPA, if measured in this cohort, may have shown additional impact on the health endpoints monitored in this study. Future studies are warranted to assess the combined effects of GLY and AMPA on reproductive and neonatal outcomes.

## Conclusion

These data, combined with our previous GLY study, support the hypothesis that trace amounts of GLY are found in nearly every pregnant mother in the U.S. First trimester urine GLY levels were significantly correlated with reduced fetal growth as referenced by BWT%ile and with NICU admission rates in non-substance exposed pregnancies. Together, these findings are consistent with the hypothesis that fetal/maternal GBH exposure can impair fetal growth and affect neonatal health at birth.

## Electronic supplementary material

Below is the link to the electronic supplementary material.

Supplementary Material 1

## Data Availability

The datasets used and/or analyzed during the current study are available from the corresponding author on reasonable request.

## List of abbreviations

ACOG: American College of Obstetrics and Gynecology.
AMPA: Aminomethylphosphonic Acid.
ANOVA: Analysis of Variance.
ART: Artificial Reproductive Technology.
BMI: Body Mass Index.
BWT%ile: Birth Weight Percentile.
CI: Confidence Interval.
cRfD: Chronic Reference Dose.
DNA: Deoxyribonucleic Acid.
EPA: Environmental Protection Agency.
EPSPS: 5-enolpyruvylshikimate-3-phosphate synthase.
GBH: Glyphosate-based Herbicides.
GLY: Glyphosate.
IARC: International Agency for Research on Cancer.
LC-MS: Liquid Chromatography-Mass Spectrometry.
LOD: Limit of Detection.
LOQ: Limit of Quantification.
OFFHS: Ontario Farm Family Health Study.
OR: Odds Ratio.
NAS: Neonatal Abstinence Syndrome.
NICU: Neonatal Intensive Care Unit.
QC: Quality Control.
PEES: Pregnancy Environmental Exposures Study.
SD: Standard Deviation.
SE: Standard Error.
SGA: Small for Gestational Age.
THC: delta-9-tetrahydrocannabinol (cannabis).
TIDES: The Infant Development and the Environment Study.
US: United States.
