# Supplementary material for: Glyphosate exposure in early pregnancy and reduced fetal growth: a prospective observational study of high-risk pregnancies
Source: Environ Health. 2022 Oct 11;21:95. doi: 10.1186/s12940-022-00906-3 (PMC9552485; doi:10.1186/s12940-022-00906-3)
Supplement: Supplementary file 1 — Supplementary Material 1 [file 12940_2022_906_MOESM1_ESM.docx]

**Additional file 1.doc. Supplemental Table 1. BWT%ile and NICU Admission associations with maternal and infant characteristics. This data further assesses the relationships between individual maternal and infant characteristics and BWT%ile and NICU Admissions.**

**Supplemental Table 1. Associations of BWT%ile and NICU Admission with maternal and infant characteristics.**

| **Characteristic** | **Group** | **N**  **(Total N=155)** | **BWT%ile** | | **NICU Admit** | |
| --- | --- | --- | --- | --- | --- | --- |
|  |  |  | **Mean ± SE** | **p-value ^a^** | **Frequency (%)** | **p-value ^b^** |
| ***Maternal*** | | | | | | |
| Age | ≤25 years | 49 | 0.46 ± 0.04 | 0.876 | 27 (55.1%) | 0.090 |
|  | 25-35 years | 76 | 0.48 ± 0.03 |  | 33 (43.4%) |  |
|  | >35 years | 30 | 0.48 ± 0.05 |  | 9 (30.0%) |  |
| Race/Ethnicity | White | 109 | 0.53 ± 0.03 | **<0.001** | 53 (48.6%) | 0.069 |
|  | Black | 32 | 0.26 ± 0.05 |  | 14 (43.8%) |  |
|  | Hispanic | 7 | 0.62 ± 0.11 |  | 2 (28.6%) |  |
|  | Other | 7 | 0.47 ± 0.11 |  | 0 (0.0%) |  |
| Marriage Status | Not Married | 64 | 0.46 ± 0.04 | 0.562 | 31 (48.4%) | 0.410 |
|  | Married/Cohabitation | 91 | 0.45 ± 0.05 |  | 38 (41.8%) |  |
| Education | <High School | 7 | 0.45 ± 0.05 | 0.654 | 14 (45.2%) | **0.004** |
|  | High School | 39 | 0.38 ± 0.11 |  | 7 (100.0%) |  |
|  | >High School | 78 | 0.52 ± 0.05 |  | 21 (53.8%) |  |
|  | Unknown | 31 | 0.47 ± 0.03 |  | 27 (34.6%) |  |
| Employment | Not Employed | 73 | 0.49 ± 0.03 | 0.590 | 33 (45.2%) | 0.871 |
|  | Employed | 82 | 0.46 ± 0.03 |  | 36 (43.9%) |  |
| Living in Large  Central Metro | No | 66 | 0.51 ± 0.04 | 0.205 | 28 (42.4%) | 0.652 |
|  | Yes | 89 | 0.45 ± 0.03 |  | 41 (46.1%) |  |
| Tobacco Use | No | 104 | 0.51 ± 0.03 | **0.039** | 35 (33.7%) | **<0.001** |
|  | Yes | 51 | 0.40 ± 0.04 |  | 34 (66.7%) |  |
| Caffeine Use | No | 59 | 0.46 ± 0.04 | 0.533 | 25 (42.4%) | 0.674 |
|  | Yes | 96 | 0.49 ± 0.03 |  | 44 (45.8%) |  |
| Alcohol Use | No | 136 | 0.46 ± 0.03 | 0.081 | 63 (46.3%) | 0.226 |
|  | Yes | 19 | 0.59 ± 0.07 |  | 6 (31.6%) |  |
| Opioid, THC, or Polysubstance Use | No | 105 | 0.47 ± 0.03 | 0.890 | 37 (35.2%) | **<0.001** |
|  | Yes | 50 | 0.48 ± 0.04 |  | 32 (64.0%) |  |
| High Stress | No | 114 | 0.50 ± 0.03 | 0.070 | 48 (42.1%) | 0.314 |
|  | Yes | 41 | 0.40 ± 0.05 |  | 21 (51.2%) |  |
| Hypertensive Disorder(s) | No | 98 | 0.45 ± 0.03 | 0.122 | 42 (42.9%) | 0.586 |
|  | Yes | 57 | 0.52 ± 0.04 |  | 27 (47.4%) |  |
| Diabetes | No | 115 | 0.44 ± 0.03 | **0.012** | 49 (42.6%) | 0.418 |
|  | Yes | 40 | 0.58 ± 0.05 |  | 20 (50.0%) |  |
| Delivery BMI | ≤25 | 21 | 0.39 ± 0.06 | 0.225 | 12 (57.1%) | 0.195 |
|  | 25-30 | 40 | 0.42 ± 0.05 |  | 19 (47.5%) |  |
|  | 30-35 | 50 | 0.52 ± 0.04 |  | 24 (48.0%) |  |
|  | 35-40 | 25 | 0.48 ± 0.06 |  | 6 (24.0%) |  |
|  | >40 | 19 | 0.56 ± 0.07 |  | 8 (42.1%) |  |
| C-Section | No | 80 | 0.44 ± 0.03 | 0.100 | 30 (37.5%) | 0.069 |
|  | Yes | 75 | 0.52 ± 0.03 |  | 39 (52.0%) |  |
| ***Infant*** | | | | | | |
| Sex | Female | 78 | 0.45 ± 0.03 | 0.373 | 35 (44.9%) | 0.929 |
|  | Male | 77 | 0.50 ± 0.03 |  | 34 (44.2%) |  |
| Birth Defects | No | 135 | 0.48 ± 0.03 | 0.718 | 54 (40.0%) | **0.003** |
|  | Yes | 20 | 0.45 ± 0.07 |  | 15 (75.0%) |  |
| Preterm Birth | <37 weeks | 32 | 0.45 ± 0.03 | **0.035** | 42 (34.1%) | **<0.001** |
|  | ≥37 weeks | 123 | 0.57 ± 0.05 |  | 27 (84.4%) |  |
| Neonatal Abstinence Syndrome (NAS) | No | 131 | 0.50 ± 0.03 | **0.039** | 45 (34.4%) | **<0.001** |
|  | Yes | 24 | 0.36 ± 0.06 |  | 24 (100.0%) |  |

^a^ P-values were obtained from one-way fixed effect (or ANOVA) models.

^b^ P-values were obtained from logistic regression models.
